# Aids to Materia Medica

**Published:** 1902-12

**Authors:** 


**Aids to Materia Medica.** By WILLIAM MURRELL, M.D. Three Parts. Pp. 91; 133; 94. London: Baillière, Tindall & Cox. 1898—1900.

*In primis* the reviewer begs to apologise to the author and publishers for the delay which an accidental circumstance has caused in the appearance of this notice of a most useful and commendable work.

In Part I. we have a complete and excellent synopsis of the uses and actions of the official inorganic *materia medica*; brief but clear statements about such accessory methods of treatment as clothing, baths, bleeding, and such like, and, in addition, a consideration of many general topics, such as prescribing, the alterations in the last edition of the *British Pharmacopœia*, and other matters.

Part II. is devoted to the consideration of drugs of vegetable origin. These are adequately but not too fully treated for the purposes of the medical student. The English language is not very well adapted to precise expression, and we have noticed some important sentences which are not devoid of equivocal meaning, caused perhaps by too great a desire to administer the instruction in the *materia medica*, "like pills, in the smallest possible compass," as the author humorously remarks in the preface. Is it the experience of most practitioners, that strophanthus "produces no gastric disturbance?" It certainly acts as an aperient when administered for some few days successively.

Part III. gives an excellent and sufficiently full account of synthetical products, drugs of animal origin, and serum therapeutics. We would desire to speak very highly of this section of the work; it is most attractively and pleasantly written, and tells all that a student needs to know of this branch of *materia medica*, both official and extra-official. We perused the book with advantage, and wish every student would do the same, and so mitigate the present deplorable ignorance on the part of medical students about drugs and all other non-surgical therapeutical measures. Does *sweet briar* form to the Englishman's mind an accurate translation of "*Betula lenta*" (p. 26)?

